# My Child Redeems My Broken Dreams: On Parents Transferring Their Unfulfilled Ambitions onto Their Child

**DOI:** 10.1371/journal.pone.0065360

**Published:** 2013-06-19

**Authors:** Eddie Brummelman, Sander Thomaes, Meike Slagt, Geertjan Overbeek, Bram Orobio de Castro, Brad J. Bushman

**Affiliations:** 1 Department of Psychology, Utrecht University, Utrecht, The Netherlands; 2 School of Psychology, University of Southampton, Southampton, United Kingdom; 3 Department of Psychology, The Ohio State University, Columbus, Ohio, United States of America; 4 Department of Communication Science, VU University Amsterdam, Amsterdam, The Netherlands; The University of Queensland, Australia

## Abstract

From the early days of psychology, theorists have observed that parents sometimes transfer their own unfulfilled ambitions onto their child. We propose that parents are especially inclined to do so when they see their child as part of themselves, more so than as a separate individual. When parents see their child as part of themselves, their child’s achievements may easily come to function as a surrogate for parents’ own unfulfilled ambitions. In the present experiment, 73 parents (89% women, *M*_age_ = 43 years) were randomly assigned to reflect on either their own or others’ unfulfilled ambitions. Results showed that, when faced with their own unfulfilled ambitions, parents who see their child as part of themselves want their child to fulfill their unfulfilled ambitions. This study provides the first experimental evidence to suggest that parents may desire their child to redeem their broken dreams.

## Introduction

From the early days of psychology, theorists have noted that parents are sometimes inclined to transfer their own unfulfilled ambitions onto their child. For example, Freud [1] noted that many parents feel that “the child shall fulfil those wishful dreams … which they never carried out” (p. 91). Similarly, Jung [2] observed that parents often desire their child “to compensate for everything that was left unfulfilled in [their own] lives” (p. 189). To be sure, these ideas are not just relics of an ancient past. Contemporary parenting experts similarly suggest that parents sometimes see their child as an object of their own unfulfilled dreams and ambitions (e.g., [3]–[5]). Surprisingly, this hypothesis has never been tested. The present research provides its first empirical test.

Unfulfilled ambitions, like other lost opportunities, are what people typically regret most in life [6], and people often try to reframe their unfulfilled ambitions to make them less painful (cf. [7]). One way for parents to reframe their unfulfilled ambitions, we suggest, is through their children. Symbolic self-completion theory [8], [9] holds that when people feel they have not fulfilled their ambitions, they often seek out symbols that complete their identities as “successful” individuals (e.g., cheering for a successful basketball player when one has failed in becoming one oneself). Parents may come to see their children as such symbols for their own success, and desire them to fulfill the ambitions they once held for themselves. Parents may feel that in their children their own unfulfilled ambitions can yet come true.

Of course, not all parents are inclined to transfer their unfulfilled ambitions onto their child. We propose that parents are especially inclined to do so when they see their child as part of themselves, more so than as a separate individual. A long tradition in psychology has held that people often incorporate close others, especially their children, into the self (e.g., [10]–[12]; for empirical evidence, see [13]–[15]). When people incorporate others into the self, they experience a sense of oneness with them–“a sense of shared, merged, or interconnected personal identities” ([16], p. 483). They experience the others’ traits and behaviors as partially their own, and they experience the world partially from the others’ point of view [13], [16], [17]. Consequently, when parents see their child as part of themselves, they may experience the child’s achievements as if they were their own (cf. [13], [18]–[20]). When these parents feel they have not fulfilled their own ambitions, their child’s achievements may easily come to function as a surrogate (cf. [21]). Thus, when parents see their child as part of the self and feel they have not lived up to their ambitions, they may be particularly likely to transfer their unfulfilled ambitions onto their child–in an attempt to fulfill through their child the dreams and ambitions they once held for themselves.

### Present Research

The present research tested this hypothesis. To manipulate the feeling of not having fulfilled one’s ambitions, parents were randomly assigned to reflect on either their own unfulfilled ambitions (experimental condition) or others’ unfulfilled ambitions (control condition). We expected that, when faced with their own unfulfilled ambitions, parents would transfer these ambitions onto their child, but only to the extent that they see the child as part of the self. We studied parents of children aged 8 to 15, because this is a time when pronounced individual differences exist in the extent to which parents perceive their children as part of the self [22], [23].

## Method

Participants were 73 parents (89% mothers) from the Netherlands of a child aged 8 to 15 (53% girls) recruited online (see [24], for a review of the representativeness of online samples). On average, participants were 43 years old (range = 31–54, *SD* = 4.5), had received 12 years of education (range = 4–18, *SD* = 3.2), and had 2 to 3 children (range = 1–4, *M* = 2.4, *SD* = 0.9). Participants were informed about the study procedures, and voluntarily subscribed for participation. In accordance with the Dutch code of conduct for scientific practice [25], no written consent was obtained because the research was non-invasive, participants had voluntarily subscribed for participation, and data were analyzed anonymously. The research ethics committee of the faculty of Social and Behavioral Sciences of Utrecht University approved all procedures.

**Figure 1:**
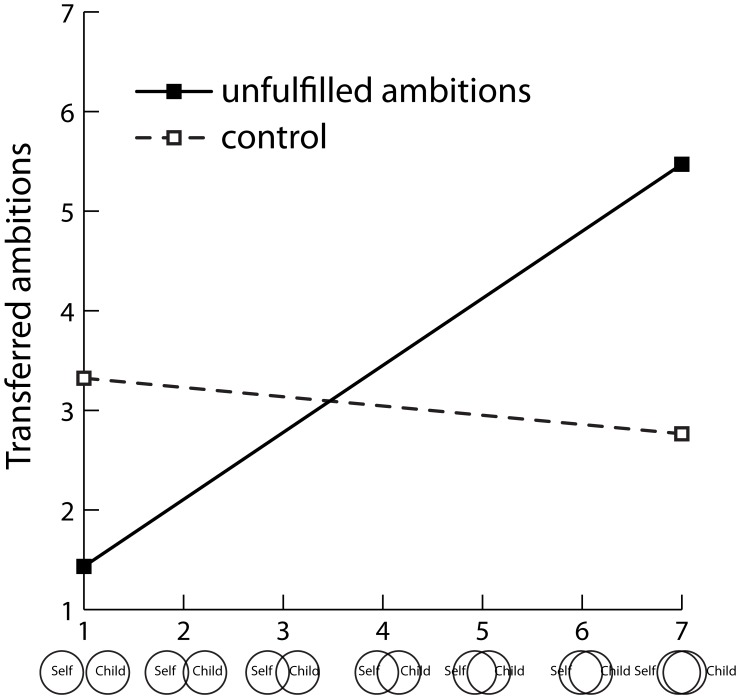
Transferred ambitions as a function of unfulfilled ambitions and inclusion of child in the self. The Inclusion of Other in the Self Scale [26] is displayed on the x-axis. Parents were instructed to select the figure that best describes their relationship with their child. Copyright © 1992 by the American Psychological Association. Adapted with permission. The official citation that should be used in referencing this material is Aron, Aron, and Smollan (1992). No further reproduction or distribution is permitted without written permission from the American Psychological Association.

In the experiment proper, participants first completed the Inclusion of Other in the Self Scale ([26], see Figure 1), a well-established pictorial scale assessing the extent to which people see another person (i.e., their child) as part of themselves (1 = *not at all*, 7 = *completely*; *M* = 3.62, *SD* = 1.27). Although this scale consists of a single item, previous research has shown that it has high test-retest stability, is unrelated to social desirability, predicts relationship quality, and has similar correlates across relationship-types (e.g., parent-child and romantic relationships; [13], [26]).

Next, participants were randomly assigned to experimental (*n* = 32) or control (*n* = 41) conditions. In the experimental condition, participants listed two ambitions they had not been able to achieve during their lives, and wrote about why these ambitions were important to them. In the control condition, participants listed two ambitions their acquaintances had not been able to achieve during their lives, and wrote about why these ambitions were important to their acquaintances. This manipulation ensured that effects can be attributed to thinking about one’s own unfulfilled ambitions in particular, rather than to thinking about unfulfilled ambitions in general.

Finally, participants reported *transferred ambitions*, which is the desire that their child will fulfill their unfulfilled ambitions (“I hope my child will…” *achieve what I wasn’t able to achieve, reach goals that I wasn’t able to reach, realize ambitions that I wasn’t able to realize, fulfill dreams that I wasn’t able to fulfill*; 1 = *disagree strongly*, 7 = *agree strongly*; *M* = 3.18, *SD* = 1.66, Cronbach’s α = .96; this instrument was developed for the present study; see Text S1 for additional information on its validity).

## Results

Inclusion of child in the self did not differ between conditions (*p* = .329), indicating successful random assignment. Data were analyzed using hierarchical regression, with transferred ambitions as dependent variable. Condition (0 = control, 1 = experimental) and inclusion of child in the self were entered in Step 1, and their interaction in Step 2. Inclusion of child in the self was centered, and the interaction term was the product of condition and centered inclusion of child in the self [27]. Neither predictor interacted with children’s gender or age, nor with parents’ gender, age, educational level, or number of children, *p*s >.127.

No main effects emerged for condition or inclusion of child in the self, *R* ^2^ = .03, *p* = .340. As predicted, an interaction emerged between condition and inclusion of child in the self, Δ*R* ^2^ = .08, *b* = 0.77, *SE* = 0.31, *p* = .015, β = .37 (see Figure 1). Simple slopes analysis revealed that inclusion of child in the self strongly predicted increased transferred ambitions when parents had reflected upon their own unfulfilled ambitions, *b* = 0.67, *SE* = 0.24, *p* = .006, β = .51, but *not* when they had reflected upon acquaintances’ unfulfilled ambitions, *b* = –0.09, *SE* = 0.19, *p* = .633, β = –.07. Similarly, simple contrasts revealed that reflecting upon their own (compared to acquaintances’) unfulfilled ambitions increased transferred ambitions among parents high (*M*+1 *SD*) in inclusion of child in the self, *t*(69) = 2.04, *p* = .045, β = .33, but *not* among parents average or low (*M* –1 *SD*) in inclusion of child in the self, *t*(69) = 0.30 and –1.55, respectively, *p*s >.126.

## Discussion

The idea that parents may transfer their unfulfilled ambitions onto their children has a long history both in the field of psychology and within popular culture. The present study is the first to test this idea, and shows that parents indeed desire their child to fulfill their unfulfilled ambitions, but only to the extent that they see their child as part of themselves. Consistent with symbolic self-completion theory [8], [9], this occurred only after parents were faced with their own unfulfilled ambitions. In the control condition, inclusion of child in the self and transferred ambitions were unrelated. Bridging classical theories on the self [9], [11] and parenthood [1], [3], these findings indicate that when parents see their children as part of the self, they may attempt to fulfill through them the ambitions they were unable to fulfill themselves.

These findings indicate that parents sometimes desire their child to become successful in precisely those domains in which they have failed themselves. At first sight, this seems at odds with the self-evaluation maintenance model [28], [29], which holds that people feel threatened (rather than proud) when a close other, such as their child, performs well in a self-relevant domain. However, more recent research suggests that when this close other is incorporated into the self, people do take pride in the other’s successes, even when these successes occur in a self-relevant domain [18], [20]. Thus, incorporating children into the self may allow parents to bask in children’s reflected glory.

Our findings provide novel insight into the psychological benefits of parenthood. Parents generally experience more meaning in life than non-parents do [30], [31], but little is known about *how* parents derive meaning from parenthood. Our research may solve one part of this puzzle: Parents may derive meaning from parenthood by vicariously resolving their unfulfilled ambitions through their children. Basking in children’s reflected glory, parents’ feelings of regret and disappointment about their own lost opportunities may gradually resolve, and make way for pride and fulfillment.

To be sure, our study does not speak to the adaptive or maladaptive value of seeing children as part of the self. Previous work has found that when people perceive others to be included in the self, their relationships with them tend to be closer and more satisfying [13], [26]. Yet, in the context of parent-child relationships, perceiving others (i.e., children) as included in the self may have potential downsides. In particular, when the perceived parent-child overlap is so strong that parents fail to differentiate between their own and their children’s perspectives (called “enmeshment”), children may have difficulty establishing an autonomous identity [32], [33].

Our findings identify new research directions. One interesting question is whether children’s accomplishments actually make parents feel more successful themselves. Symbolic self-completion theory suggests that people feel successful especially when their symbols of success (in this case, their children’s successes) are noticed by others [34]. This suggests that parents might sometimes feel inclined to showcase their children’s accomplishments to the outside world, in an attempt to bolster their own feelings of success. Another question is whether the present findings extend beyond parent-child relationships. Because people readily incorporate close others in the self, regardless of whether they are in vertical (e.g., parent-child) or horizontal (e.g., romantic) relationships [11], our findings may represent a universal relationship mechanism to deal with unfulfilled ambitions. However, ambition transference might be especially likely to occur in parent-child relationships, for two reasons. First, the parent-child relationship cannot dissolve. Regardless of the quality of the relationship, parents always hold a genetic tie to their children [35]. Second, compared to other relationship partners (e.g., romantic partners and friends), children typically have more time and opportunities ahead of them to fulfill their parents’ unfulfilled ambitions.

Our study is not without limitations. First, its sample consisted mainly of mothers. Because mothers may be more inclined than fathers to incorporate their children into the self [36], ambition transference may be more prevalent among mothers than fathers. Second, our study focused on parents’ *desire* that their child will fulfill their unfulfilled ambitions. Future research should examine how this desire translates into actual parenting practices (e.g., attempts to influence children’s career choices). Third, because the transferred ambitions measure was developed for this study, evidence on its validity is currently limited. Future research needs to corroborate its validity.

### Conclusions

Unfulfilled ambitions hurt many of us. An important task for psychologists is to identify how people deal with such disappointments. The present research has identified a striking way in which some parents deal with their unfulfilled ambitions, namely by transferring them onto their successor–their child. Parents may thus desire their child to redeem their broken dreams.

## Supporting Information

Text S1 **Additional information on the validity of the transferred ambitions measure.**
(DOC)
