# Supplementary material for: My Child Redeems My Broken Dreams: On Parents Transferring Their Unfulfilled Ambitions onto Their Child
Source: PLoS One. 2013 Jun 19;8(6):e65360. doi: 10.1371/journal.pone.0065360 (PMC3686790; doi:10.1371/journal.pone.0065360)
Supplement: Text S1 — Additional information on the validity of the transferred ambitions measure. (DOC) [file pone.0065360.s001.doc]

Supporting Information S1

Symbolic self-completion theory suggests that people feel successful especially when their symbols of success are noticed by others [1]. Consequently, when parents transfer their unfulfilled ambitions onto their children and thus use children’s successes as symbols of their own success, they may become particularly concerned with obtaining external approval of children’s successes. We therefore propose that transferred ambitions are extrinsic in nature (i.e., concerned with obtaining external approval, such as becoming rich and famous), rather than intrinsic (i.e., satisfying in their own right, such as developing love and self-knowledge; [2]).

To test this, participants also completed a shortened, 12-item version of the Aspiration Index [3]. They were presented prototypical extrinsic aspirations (*to be financially successful, to be rich, to be admired by lots of different people, to be famous, to keep up with fashions in hair and clothing, to have an image that others find appealing*) and intrinsic aspirations (*to grow and learn new things, to know and accept who [s]he really is, to have good friends that [s]he can count on, to have committed, intimate relationships, to work for the betterment of society, to help people in need*). They rated how important it is to them that *their child* will achieve each aspiration. Responses were given on 7-point scales (1 = *not at all important,* 7 = *very important*) and averaged across items for extrinsic aspirations (Cronbach’s α = .73, *M* = 2.89, *SD* = 0.84) and intrinsic aspirations (Cronbach’s α = .79, *M* = 6.05, *SD* = 0.59).

Pearson correlations showed that, collapsed across conditions, transferred ambitions were associated with parents’ extrinsic aspirations (*r*[71] = .36, *p* = .002) but *not* intrinsic aspirations (*r*[71] = –.05, *p* = .705) for their child. These links did not differ between conditions, *p*s > .971. Thus, when parents transfer their unfulfilled ambitions onto their child, they do not simply want their child to succeed; instead, they specifically want their child to succeed in achieving extrinsic ambitions.

Supplementary References

1. Gollwitzer PM (1986) Striving for specific identities: The social reality of self-symbolizing. In: Baumeister R, editor. Public self and private self. New York: Springer-Verlag. pp. 143-159.

2. Ryan RM, Deci EL (2000) Self-determination theory and the facilitation of intrinsic motivation, social development, and well-being. Am Psychol 55: 68-78.

3. Kasser T, Ryan RM (1993) A dark side of the American dream: Correlates of financial success as a central life aspiration. J Pers Soc Psychol 65: 410-422.
